# Treatment of Cystic Fibrosis: From Gene- to Cell-Based Therapies

**DOI:** 10.3389/fphar.2021.639475

**Published:** 2021-03-16

**Authors:** Katelin M. Allan, Nigel Farrow, Martin Donnelley, Adam Jaffe, Shafagh A. Waters

**Affiliations:** ^1^ School of Women’s and Children’s Health, Faculty of Medicine, University of New South Wales, Sydney, Australia; ^2^ Molecular and Integrative Cystic Fibrosis Research Centre (miCF_RC), University of New South Wales and Sydney Children’s Hospital, Sydney, Australia; ^3^ Respiratory and Sleep Medicine, Women’s and Children’s Health Network, Adelaide, Australia; ^4^ Robinson Research Institute, The University of Adelaide, Adelaide, Australia; ^5^ Adelaide Medical School, The University of Adelaide, Adelaide, Australia; ^6^ Department of Respiratory Medicine, Sydney Children’s Hospital, Sydney, Australia

**Keywords:** cystic fibrosis, CFTR, gene therapy, cell-based therapy, therapeutic vectors, stem cells

## Abstract

Prognosis of patients with cystic fibrosis (CF) varies extensively despite recent advances in targeted therapies that improve CF transmembrane conductance regulator (CFTR) function. Despite being a multi-organ disease, extensive lung tissue destruction remains the major cause of morbidity and mortality. Progress towards a curative treatment strategy that implements a *CFTR* gene addition-technology to the patients’ lungs has been slow and not yet developed beyond clinical trials. Improved delivery vectors are needed to overcome the body’s defense system and ensure an efficient and consistent clinical response before gene therapy is suitable for clinical care. Cell-based therapy–which relies on functional modification of allogenic or autologous cells *ex vivo*, prior to transplantation into the patient–is now a therapeutic reality for various diseases. For CF, pioneering research has demonstrated proof-of-principle for allogenic transplantation of cultured human airway stem cells into mouse airways. However, applying a cell-based therapy to the human airways has distinct challenges. We review CF gene therapies using viral and non-viral delivery strategies and discuss current advances towards autologous cell-based therapies. Progress towards identification, correction, and expansion of a suitable regenerative cell, as well as refinement of pre-cell transplant lung conditioning protocols is discussed.

## CFTR Correction Strategies

Cystic fibrosis (CF) is an inherited, multi-organ disease caused by mutations in the CF transmembrane conductance regulator (*CFTR*) gene (Rowe et al., 2005). CF is progressive, with its major pathology impacting the lung, liver, pancreas and intestine. Mortality in CF patients is mostly due to respiratory failure (Elborn, 2016). The CFTR protein, an anion channel, is expressed in a diverse range of epithelial tissues (Riordan, 2008). CFTR dysfunction disrupts ion transport equilibrium, deregulating fluid absorption and secretion processes in epithelial tissue such as the airways, resulting in mucus accumulation and recurrent bacterial infections (Ratjen et al., 2015). Symptomatic therapies such as airway clearance by physiotherapy, mucus thinning agents, antibiotics and anti-inflammatories remain crucial for the management of CF airways (Flume et al., 2007).

Recently, targeted therapies have been approved for CF treatment. These small molecule compounds modulate CFTR protein abundance and/or function at the apical epithelial cell membrane (Davies, 2015). A combination of three CFTR modulating small molecules–elexacaftor/tezacaftor/ivacaftor–is the most advanced targeted therapeutic approved for patients with one or two Phe508del-CFTR alleles–the most common *CFTR* mutation in the population. CFTR modulator therapies result in improved lung function and better quality of life for patients with CF (Davies et al., 2018). Yet, variable therapeutic response, inadequate long-term efficacy data, adverse effects and unavailability of modulators for the 10% of CF patients with mutations that produce little or no CFTR protein has rekindled great interest in the development of *CFTR* mutation-independent corrective strategies.

*In vivo* transfer of a functional copy of *CFTR* has been envisioned as a CF airway treatment since 1989 when the *CFTR* gene was identified as the cause of this multisystemic disease (Tsui et al., 1985; Wainwright et al., 1985). Gene therapy has received FDA approval for treatment of monogenic disorders (U.S. Food and Drug Administration. 2020) such as spinal muscular atrophy (Kariyawasam et al., 2018), coagulative disorders (Batty and Lillicrap 2019), and immunodeficiency diseases (Booth et al., 2019), but not yet for CF. Numerous research programs and clinical trials have been undertaken to explicate the most effective vector (viral or non-viral) to deliver *CFTR* to airway cells (Griesenbach et al., 2015). However, clinical efficacy of these vectors *in vivo* in humans has been insignificant and inconsistent in improving lung function (Alton et al., 2015a). The greatest barrier to enabling clinical translation of gene therapy for CF remains the lack of an effective delivery system to the lungs. A successful gene therapy system for restoration of CFTR function needs to navigate the complexities of the lung clearance and innate immunity defense functions that are further complicated in the CF airways due to increased mucus volume and viscosity (reviewed in (Donnelley and Parsons 2018)). Even if these obstacles are circumvented, heterogeneous and highly regulated CFTR expression in various cell types of the lung raises the question of the most appropriate cellular target.

**FIGURE 1:**
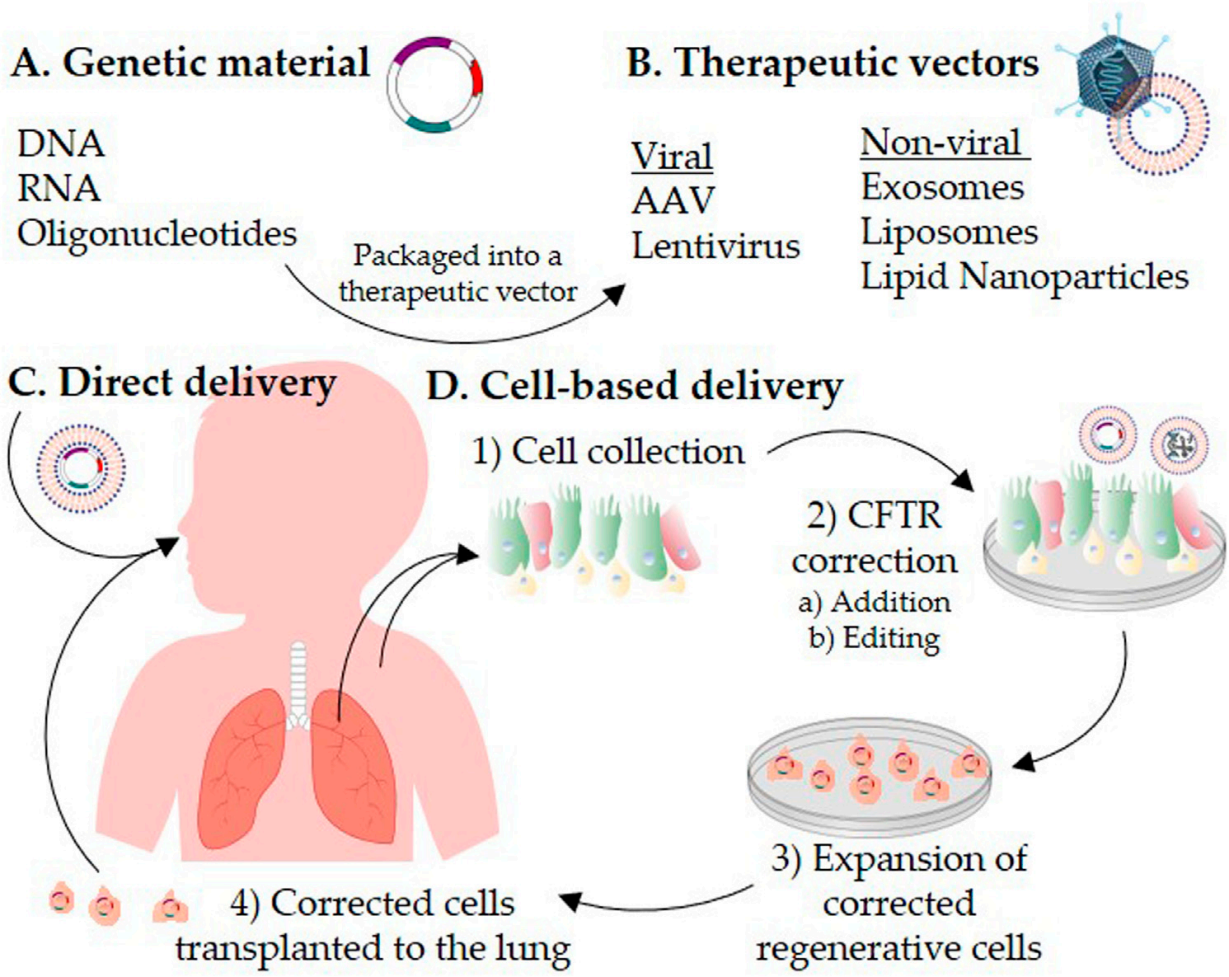
Schematic representation of CFTR correction strategies for the treatment of cystic fibrosis. Genetic materials **(A)** are packaged into a therapeutic vector **(B)**. The therapeutic vector is delivered directly to the patient’s lungs **(C)** or introduced into cells *ex vivo* **(D)**. For autologous cell-based therapy, 1) airway cells are isolated from the patient’s respiratory tract or induced pluripotent stem cells are generated from the patient’s skin fibroblasts or blood. 2) CFTR is corrected *in vitro* in the collected cells by a) addition or b) editing strategies. 3) The CFTR-corrected regenerative cells are expanded to reach a therapeutic dose, and then 4) transplanted back to repopulate the patient lung epithelium.

One proposed strategy to deal with the challenges associated with *in vivo* delivery of *CFTR* to the airway cells is to correct the airway cells *ex vivo* followed by transplanting the corrected cells to repopulate the patient’s lung with *CFTR*-corrected cells (Figure 1). This approach is the basis of the first *ex vivo* hematopoietic stem cell gene therapy, Strimvelis, which was approved for treatment of adenosine deaminase-severe combined immunodeficiency (Stirnadel-Farrant et al., 2018). In this review, we will first describe alternative strategies to CFTR DNA therapy, and discuss the advances in the main groups of viral and non-viral vectors that have shown promise in CF therapy. The second part of this review will focus on recent progress in cell-based therapies, including the gene editing technologies that facilitate CFTR correction in *ex vivo* cells*.*

## Therapeutic Genetic Material Other Than DNA: RNA Addition and Repair

The earliest efforts to deliver genetic material into diseased cells focused on directly introducing therapeutic *CFTR* DNA as an addition strategy to subsequently produce functional CFTR protein (reviewed in (Cooney et al., 2018)). A novel alternative to DNA therapeutics is based on addition of RNA. Since the functional site of messenger RNA (mRNA) is the cell cytoplasm, the challenge of nuclear translocation is eliminated (Hajj and Whitehead 2017).

Exogenous nucleic acids are susceptible to degradation by nucleases and can trigger an immune response upon cellular entry (Alexopoulou et al., 2001; Kariko et al., 2004). Therefore, current strategies utilize chemical modification of the nucleic acid bases to reduce immunogenicity and increase stability (Sahin et al., 2014; Pardi et al., 2015). Manufacturing and addition of modifications to RNA is easier than DNA, extending the usefulness of RNA therapy (Kuhn et al., 2012). Yet, repeat RNA administration remains necessary to sustain therapeutic levels of protein (Patel et al., 2019b). Successful delivery of chemically modified CFTR mRNA to patient-derived bronchial epithelial cells has demonstrated increased CFTR expression at the plasma membrane and rescue of chloride transport (Robinson et al., 2018). Aerosolized delivery of *in vitro* transcribed CFTR mRNA complexed with lipids to the lungs (Orphan drug MRT5005) is a therapeutic forerunner currently being tested in a phase 1/2 trial (NCT03375047) which has highly anticipated results for *in vivo* correction of CFTR.

In addition to direct RNA supplementation, an alternative therapeutic option is to alter the defective RNA to restore proper CFTR protein function. This can be achieved using antisense oligonucleotides (ASOs). ASOs are short synthetic oligonucleotides that are chemically modified to bind to target RNA, offering a tool for direct mRNA restoration (Oren et al., 2017). *In vivo* delivery of ASOs to the airway epithelium of mice initially resulted in no significant uptake (Griesenbach et al., 2006). It was postulated that this inefficiency was due to physical barriers to airway epithelial cell delivery (Griesenbach et al., 2006). Advances in RNA chemical modifications have been key to the development of a successful ASO-mediated therapy, with the accelerated FDA approval of eteplirsen (Exondys 51) being granted in 2016 for patients with Duchenne muscular dystrophy that have a mutation in the *dystrophin* gene which can be treated by exon 51 skipping (reviewed in (Bennett et al., 2017)). An ASO candidate for ASO-directed mRNA restoration, Eluforsen, binds to and restores the mRNA region encoding the Phe508 deletion (Beumer et al., 2019). In a phase 1 study, repeated administration of Eluforsen improved CFTR activity in patients homozygous for the Phe508 mutation (Sermet-Gaudelus et al., 2019) (NCT02564354). In the following phase 1b safety study with 70 enrolled participants Eluforsen was well-tolerated (NCT02532764), supporting the therapeutic translation of novel mRNA-based therapies for patients with CF. ASOs have also been tested for their ability to correct *CFTR* splicing mutations, which constitute 13% of disease-causing *CFTR* mutations, and result in no functional protein being produced (Igreja et al., 2016). Single-stranded DNA oligonucleotides designed to hybridize to pre-mRNA and modify aberrant splicing restored splicing in immortalized cells expressing the *CFTR* splicing mutation, c.2657 + 5G > A-*CFTR* (Igreja et al., 2016).

Although clinical translation of DNA or RNA therapy has been proven feasible, the efficient targeting and delivery of these genetic materials into the lung epithelium for treatment of CF remains exceedingly challenging.

## Therapeutic Delivery Vectors

Nucleic acids are unable to cross the cell membrane because of their high negative charge, and therefore require assistance for intracellular delivery (reviewed in (Ni et al., 2019)). Numerous viral and non-viral delivery systems have been investigated for their ability to transfer genetic material–RNA (Therapeutic Genetic Material Other Than DNA: RNA Addition and Repair), DNA (this section) and gene editing system components (Gene Editing Technologies)–to the lung. The main groups of viral and non-viral vectors used in CF therapy and advances toward their clinical translation will be summarized below.

### Viral Vectors for Airway Gene Therapy

Viral vector-mediated gene delivery systems take advantage of a virus’s natural ability to evade the lung mucus barrier and transduce the airway by inserting their DNA into epithelial cells. The natural tropism of viral vectors enables preferential targeting of the airway progenitor cells–the basal cells–so that multi-lineage transgene expression can be achieved (Havenga et al., 2002). Other advantages and disadvantages of available vectors have been discussed in depth elsewhere (Miah et al., 2019). Here we briefly discuss the most commonly used vectors for CF.

#### Adeno-Associated Virus

In the early 2000s, human CF clinical trials tested aerosolized viral vectors, including AAV serotype 2 (AAV2). Based on delivery of CFTR complementary DNA (cDNA) to the human nasal cavity and lung, AAV was determined safe, but failed to demonstrate significant clinical benefit in restoration of lung function (Wagner et al., 2002; Flotte et al., 2003; Moss et al., 2004; Moss et al., 2007). These findings have been attributed to issues regarding the packaging capacity of the AAV2 genome to encompass a CFTR expression cassette (Wu et al., 2010) and also low transduction efficiency of the airway epithelial cells (AECs) due to the limited binding of AAV2 to the apical surface of human airway epithelia (Zabner et al., 2000).

To overcome the packaging capacity limitation of AAV, a split AAV gene delivery approach can be implemented, wherein a large transgene is split across multiple separate AAV vectors (Patel et al., 2019a). As a means of screening for AAV serotypes that display airway tropism and permit efficient gene delivery to the human lung parenchyma, lung bud organoids–a model of lung parenchyma derived from human embryonic stem cells–have been utilized (Meyer-Berg et al., 2020). A 2020 study identified AAV2 and AAV serotype 6 (AAV6) as having the highly efficient transduction of the human lung parenchyma. Additionally, it was recently discovered that unlike other AAV serotypes, AAV6 is capable of efficiently diffusing through mucus in primary differentiated CF airway cultures (Duncan et al., 2018). Inhaled administration of AAV6 provided high-level transgene expression (mediating roughly 30% airway coverage) in a mouse model with airway mucus obstruction (Duncan et al., 2018). Yet, the high immunogenicity of AAV draws concern to the potential for participants of viral-based gene therapy clinical trials to acquire AAV immunity, and for potential preexisting immunity in some patients (reviewed in (Ronzitti et al., 2020). These are major concerns because immunity to AAV is likely to prevent repeat administration of AAV gene therapy to patients (George et al., 2020), therefore risking their eligibility for future AAV-based therapies. In 2020, the imlifidase (IdeS) enzyme was tested in rodents and non-human primates and successfully demonstrated inhibition of the host immune response to AAV resulting from preexisting immunity and following AAV gene therapy (Leborgne et al., 2020). If IdeS treatment in human patients were to enable repeat administration of AAV-based therapies, this could have big implications for the future course of AAV gene therapy. To improve the gradual decrease in *CFTR* expression due to the transient episomal expression nature of AAV, and avoid the need for repeat dosing of the AAV vector, Cooney and colleagues designed a novel integrating AAV-based *CFTR*-expressing vector (termed *piggyBac*/AAV), which demonstrated efficient transduction and persistent expression in primary human CF airway cells *in vitro* and in mouse airways *in vivo* (Cooney et al., 2015). A follow-up large animal study delivered aerosolized *piggyBac*/AAV-CFTR to CF pig airways and demonstrated phenotypic restoration of CFTR function (Cooney et al., 2019).

#### Lentivirus

In comparison to AAVs, lentivirus vectors (LV) have a larger packaging capacity (approximately 8 kb) which makes them more compatible for full length CFTR packaging (Castellani and Conese, 2010). Additionally, LVs can transduce and integrate into the genome of both dividing and non-dividing cells (Naldini, Blomer et al., 1996; Wang et al., 1999). Proof-of-principle that a lentiviral vector could correct the CFTR defect *in vivo* and provide persistent *CFTR* expression was first demonstrated in mouse airways (Limberis et al., 2002). This was followed by a demonstration of partially restored *in vivo* CFTR channel activity following aerosol delivery of Feline immunodeficiency virus (IV)-*CFTR* to CF pig airways (Cooney et al., 2016). More recently, insertion of a LacZ marker gene into airway basal cells via a LV vector produced persistent transgene expression and importantly, the basal cells successfully passed on the introduced gene to their daughter cells in a mouse airway (Farrow et al., 2018b). In support of a first-in-man CF trial, a simian IV-based lentiviral vector, pseudotyped with Sendai virus fusion protein and Hemagglutinin/Neuraminidase envelope proteins exhibited efficient transduction of human airway cells *in vitro* and murine lung epithelium *in vivo* (Alton et al., 2017).

### Non-viral Vectors

Progression of viral vectors as a clinical therapy for CF remains contingent on demonstrating successful long-term transduction efficacy, the safety of delivery to the CF lung and the ability for repeat dosing (Donnelley and Parsons, 2018). Some of these barriers have necessitated the development of more cost-effective non-viral vectors to provide an increased safety profile and limitless genetic material packaging size, while obtaining more reproducible delivery outcomes. Non-viral vectors are naturally produced by the body and can also be engineered in the laboratory. Despite reduced immunogenicity, the transfection efficiency of non-viral vectors is still low compared to viral vectors. Specifically, unmodified non-viral vectors are rapidly cleared and have low accumulation in target tissues and cells (Takahashi et al., 2013; Smyth et al., 2015), thus new compounds are constantly engineered and investigated (Murphy et al., 2019).

#### Exosomes

Exosomes are naturally occurring nanoscale extracellular vesicles which, following their release from cells, facilitate intercellular communication by transporting material to neighboring or distant recipient cells (Doyle and Wang, 2019). Due to this ability to function as an endogenous intercellular cargo transfer system, they have been exploited as vehicles for the delivery of genetic material, and as such, are a promising vector for *in vivo* gene therapy. Exosomes have been demonstrated to deliver human CFTR mature glycoprotein, as well as CFTR mRNA, in both Chinese hamster ovarian cells (Gonzalez et al., 2012) and CFTR-deficient cells derived from CF patients (Vituret et al., 2016; Villamizar et al., 2019). In both models, CFTR channel function was shown to be corrected in exosome-recipient cells. Exosomes have similarly shown utility in delivering small interfering RNA in well-differentiated human AECs (Singh et al., 2020), suggesting value beyond gene transfer for the delivery of gene editing materials. These studies demonstrated the potential application of exosomes as vectors for CFTR transfer and functional correction of the genetic defect in human CF cells.

#### Liposomes

Liposomes are artificially created vesicles with a lipid bilayer membrane and an aqueous core (Akbarzadeh et al., 2013). In a single dose, dose-escalation phase 1/2a safety trial assessment of a liposomal mixture that included the cationic lipid mixture (GL67A) complexed with *CFTR* complementary DNA, patients with CF showed improved lung function and no adverse effects (Alton et al., 2015b). This was followed by a double-blind, placebo-controlled multi-dose phase 2b trial (NCT01621867) that found that repeated administration of GL67A liposome significantly, yet modestly, stabilized lung function in the treatment group (*n* = 62) vs. placebo (*n* = 54) in CF adults or children aged 12 years or older enrolled in this trial (Alton et al., 2015a). Although this trial reached its primary efficacy endpoint (lung function improvement), the magnitude and variability in effect did not support progression to phase 3 trials. These results established for the first time a proof of principle that gene therapy was capable of favorably modulating CF lung function, with no safety concerns with repeat dosing. However, they also highlighted the need for more efficacious methods of gene delivery transport, such as viral vectors, before large-scale clinical translation can be achieved. Gene delivery systems likely face greater barriers in adults with established lung disease and irreversible fibrotic scarring in comparison to children, with less progressed lung disease. Strong reasoning to include children in gene therapy clinical trials has been given previously (Jaffe et al., 2006; Delhove et al., 2020) and may present the most rational avenue forward as a clinical target age.

#### Lipid Nanoparticles

Like liposomes, lipid nanoparticles (LNPs) are used to encapsulate and deliver therapeutic RNA formulations to the lung via intranasal or systemic routes. However, LNPs differ in their composition, often carrying cargo within a non-aqueous core (Kraft et al., 2014). Recent advances have focused on exploiting the dynamic structure of LNPs to engineer specialized formulations tailored to increase mucus penetrance (Nafee et al., 2018; Wan et al., 2018) and evade host immune detection (Vencken et al., 2019). This work climaxed in the successful LNP-mediated delivery of chemically modified CFTR mRNA to CF airway cells (Robinson et al., 2018). However, it has since been shown that LNPs only modestly increase CFTR expression in patient-derived human nasal epithelial cells (Villamizar et al., 2019). It is probable that the inefficiency of LNP delivery is due to endosome retention (Sahay et al., 2013), and as such, further work to engineer particles capable of escaping endosome retention is needed. Since CFTR expression in the epithelial airway is cell type specific (Plasschaert et al., 2018), further testing in *vitro* and *in vivo* models will be required before LNPs can be established as efficient and clinically relevant delivery systems.

## Cell-Based Therapies

Cell-based therapies offer an alternative therapeutic opportunity to *in vivo* gene therapy. This approach integrates advances in stem cell biology with the well-established platform of *in vitro* gene delivery and repair strategies. The exemplar for cell-based therapy is hematopoietic stem cell transplantation, which is successfully performed in over 50,0000 patients per year world-wide (Gratwohl and Niederwieser 2012). The safety of correcting a patient’s cells *ex vivo* via a gene editing technology, before reintroducing them into the same patient (autologous transplantation), has been demonstrated in a clinical trial for hematological malignancies (Stadtmauer et al., 2020). Autologous cell therapy has also been used for treatment of epidermolysis bullosa (Eichstadt et al., 2019; Lwin et al., 2019; Marinkovich and Tang 2019), whilst treatment of insulin-dependent diabetes has shown success with transplant of allogenic islet cells (Kumagai et al., 2002; Yamada et al., 2011).

The efficacy of these regenerative cellular therapies has propagated investigation of cell-based therapy for CF. Yet, in the CF airways, the volume, viscosity and composition of the mucus that protects the airway from foreign particles and irritants is altered in such way that penetrating this barrier poses a great challenge. Successful clinical translation of a cell therapy approach will require regenerative cell 1) identification, 2) CFTR correction (addition or editing), 3) expansion, and 4) transplantation back into the patient’s lungs (Figure 1). The remainder of this review will discuss the progress and challenges toward these milestones.

### Identification of Suitable Regenerative Cells With Differentiation Capacity

One of the obstacles to cell-based therapy for CF is the identification of an optimal self-renewing cell that can also differentiate into the cells of the airway epithelium. In this review we discuss therapeutic perspectives of using adult tissue-resident basal stem cells, yet first we will briefly describe advances in the field using mesenchymal and induced pluripotent stem cells (iPSCs).

Mesenchymal stem cells (MSCs) are an allogenic source of cells that have been investigated for their application in CF cell-based therapy due to their immunomodulatory and anti-inflammatory properties (reviewed in (Caretti et al., 2019)). In favor of their potential for CF cell therapy, MSCs co-cultured with CF immortalized airway epithelial cells at air-liquid interface have demonstrated acquisition of an epithelial phenotype, and subsequent restoration of functional CFTR protein (Carbone et al., 2014; Carbone et al., 2018). However, there is concern over low engraftment levels of administered MSCs in the lung (Ortiz et al., 2003; Xu et al., 2007). Furthermore, recent studies suggest that MSCs function transiently to reduce inflammation via the secretion of extra-cellular vesicles such as exosomes (Zhu et al., 2014; Zulueta et al., 2018) that can be used for delivery of drugs or gene editing material (reviewed in (Almeida-Porada et al., 2020)). Exosomes derived from MSCs genetically engineered to carry a transcription activator protein have demonstrated success in targeting and activating CFTR transcription in primary human bronchial epithelial cells from patients with CF (Villamizar et al., 2021). Further *in vitro* and *in vivo* studies are needed before this approach is considered viable. Until then, there remains uncertainty surrounding the capacity of MSCs to effectively restore CFTR function in the respiratory epithelium.

iPSCs are routinely generated from skin or blood cells that are reprogrammed back into an embryonic-like pluripotent state. In addition, they are amenable to gene editing and expansion, thereby providing a large potential source of gene-corrected cells which upon differentiation into lung basal stem cells can be engrafted into the lung for therapeutic use. Two iPSC-derived cell populations have been isolated for the purpose of CF cell therapy: one- airway epithelial cells (AECs) or 2-basal stem cells. The strengths and limitations of both these will be discussed here briefly. Firstly, several reports have demonstrated successful directed differentiation of human iPSCs into AECs *in vitro* (Firth et al., 2014; Huang et al., 2014; Dye et al., 2015; Konishi et al., 2016; McCauley et al., 2017). The iPSC-derived AEC cultures are a heterogenous cell population containing limited numbers of basal cells with self-renewing capacity (McCauley et al., 2017). As such, transplanting AECs will only provide a short-term solution, even if engraftment of cells was successful. Therefore, although this method can generate ample AECs, transplantation of terminally differentiated AECs would not be an effective CF cell therapy.

More recently, iPSCs have been differentiated into basal cells, termed induced basal cells (iBCs) (Hawkins et al., 2020). These iBCs are functionally indistinct from native basal cells and have been shown *in vitro* to differentiate into a pseudostratified airway epithelium exhibiting CFTR activity (Hawkins et al., 2020). However, the rarer cell types of the epithelium such as the CFTR-expressing ionocytes, and brush cells, were not observed in *vitro* cultures of iBCs differentiated at air-liquid interface (Hawkins et al., 2020). Furthermore, the long-term effects of these manipulations are not well understood, and several questions regarding the *in vivo* competence of iBCs remain unanswered. For these reasons, development of additional screening assays similar to the dual fluorescent transporter assay used by Hawkins and colleagues will be necessary to verify the cellular phenotype and differentiation capacity of iBCs in comparison to their endogenous counterparts both *in vitro* and *in vivo* before clinical translation of iPSC-based CF cell therapy can be considered viable.

To improve the chances of successful cell transplantation to the lung, it is possible that tissue-resident adult stem cell from the lung itself would be a better suited candidate. For some time, there had been a lack of clarity as to the identity of airway stem cells. However, increased knowledge of stem cell biology together with the characterization of lung progenitor lineage has brought emerging consensus that basal cells are a stem cell or progenitor cell of the airway epithelium (Rock et al., 2009; Bertoncello, 2016). Importantly, basal cells have the capacity to differentiate into all the cell types of the pseudostratified airway epithelium, including the rare population of CFTR high-expressing ionocytes (Montoro et al., 2018; Plasschaert et al., 2018). This means that upon CFTR correction of basal cells, multi-lineage expression of the corrected CFTR could be achieved in the airway epithelium.

The existence of multiple progenitors with various differentiation capacities has been detected in human bronchial xenografts (Engelhardt et al., 1995). This is in concordance with reports that mouse tracheal basal cells comprise two molecularly distinct subpopulations of multipotent stem cells and committed secretory precursors (Hong et al., 2004; Watson et al., 2015). More recently, a single-cell RNA-seq study on human airway epithelial cells identified a heterogenous population of basal cells that include multipotent and secretory-primed subsets (Carraro et al., 2020). However, it remains unknown whether secretory-primed basal cells represent a transitory state of basal cells or a phenotypically stable state. The implication is that cell therapy approaches will have to account for basal cell subtypes when selecting a suitable cell to target.

### Correcting CFTR in Regenerative Cells

Correction of CFTR *in vitro* in airway epithelial cells can be achieved by direct addition of genetic material as discussed in the sections above (Therapeutic Genetic Material Other Than DNA: RNA Addition and Repair and Therapeutic Delivery Vectors). An alternative to introducing new material is to correct the defective *CFTR* via gene editing.

#### Gene Editing Technologies

The advent of gene editing technologies has refueled excitement over gene therapy and is an area of vast development. The three major gene editing technologies are clustered regularly interspaced short palindromic repeat (CRISPR)–Cas-associated nucleases, programmable nucleases, such as zinc-finger nucleases (ZFNs) and transcription activator-like effector nucleases (TALENs). The first *in vivo* application of CRISPR/Cas9 was employed for a person with a mutation in the *CEP290* gene which causes retinal degeneration (Ledford, 2020). Clinical trials are also underway to evaluate these therapeutic approaches for treatment of cancer and sickle cell disease (Ernst et al., 2020). In the context of CF, emerging gene editing technologies hold the potential to repair specific *CFTR* gene mutations and restore their function, offering the ultimate opportunity for precision medicine. However, to date, gene editing technologies for CF remain in the preclinical realm.

The first study of CRISPR/Cas9 as a potential therapy for CF used site-specific knock-in of the correct *CFTR* sequence to robustly restore CFTR function in human intestinal stem cell organoids derived from patients homozygous for the Phe508del mutation (Schwank et al., 2013). This approach has since been implemented in cell culture by Ruan and colleagues to achieve greater than 20% repair of patient-derived induced pluripotent stem cells (iPSCs) (Ruan et al., 2019). Furthermore, in recent advancements, an AAV-delivered Cas9 gene editing platform facilitated correction of >30% Phe508del-*CFTR* in patient-derived airway basal cells, prior to transplantation to an *ex vivo* engraftment scaffold and near-normal levels of CFTR function were restored (Vaidyanathan et al., 2020). Considering reports that suggest as little as 4.7% of WT CFTR expression can lead to a milder CF phenotype (Ramalho et al., 2002), prospects for clinical translation are promising. However, a shortcoming of these gene editing technologies is that they are mutation specific. As such, different mutations must be individually corrected, with some being more amenable to this strategy than others.

CRISPR/Cas9 has successfully corrected *CFTR* splicing mutations (Sanz et al., 2017) and mutations leading to the formation of a premature termination codon that produce no functional CFTR protein (Erwood et al., 2020). As such, an approach for allele-specific editing has been outlined. Gene editing using Zinc finger nucleases (ZFNs) has also facilitated the correction of iPSC-derived CF AECs (Crane et al., 2015). Crane and colleagues showed that *CFTR*-corrected iPSCs, following induced differentiation *in vitro,* expressed functional CFTR protein (Crane et al., 2015). A similar study used ZFNs to correct Phe508del and demonstrated restored CFTR protein expression and function in air-liquid interface cultures established from the edited basal cells (Suzuki et al., 2020). TALEN technologies have also been used to restore normal CFTR expression and activity in organoids derived from Phe508del patient-derived iPSCs (Fleischer et al., 2020). The ability to achieve *CFTR* correction in additional stem/progenitor cells such as airway basal cells, either as primary airway basal cells or those derived from iPSCs, has specific relevance for progress toward cell-based therapies for CF.

### Expansion of CFTR-Corrected Regenerative Cells That Retain Differentiation Capacity

Irrespective of which regenerative cell type or *in vitro* correction method is to be used, cell-based therapy will require large numbers of viable cells to repopulate the lung by replacing the CFTR defective endogenous AECs. Hayes and colleagues have estimated that 60 million regenerative cells will be required to treat a human patient with cystic fibrosis cell therapy (Hayes et al., 2019). However, procurement of basal lung epithelial cells via bronchial lavage, sputum collection or endobronchial biopsy provides only low cell numbers (Mou et al., 2012; Pollock et al., 2013; Butler et al., 2016). To overcome the issue of low initial cell numbers, extensive cell expansion will be required prior to implantation.

Bronchial basal cells have a limited lifespan (Ghosh et al., 2011) with differentiation capacity that decreases over time *in vitro* (Gentzsch et al., 2017). Various culture protocols for cell expansion have been adapted to overcome these limitations (reviewed in (Awatade et al., 2018)). The conditionally reprogrammed cell (CRC) methodology (Liu et al., 2012) is one approach to increase bronchial cell yield (Martinovich et al., 2017) and maintain differentiation potential for multiple passages (Lee et al., 2020). CF and non-CF bronchial basal cells have been expanded to a therapeutic dose of 60 million cells via a modified CRC method, albeit the frequency of regenerative cells having decreased to 20% by the time the therapeutic dose was reached (Hayes et al., 2019). Importantly, the bronchial basal cells were demonstrated to retain their differentiation potential from passages 2 to 15 using this approach (Hayes et al., 2019). Yet, further concern is raised by reports of *in vitro* basal cell expansion altering CFTR functional activity (Awatade et al., 2021) and differentiation cell-fate composition, leading to a reduced number of ciliated cells in expanded cells compared to freshly isolated cells (Eenjes et al., 2018). If expanded basal cells differentiate to pseudostratified epithelium with an altered cell type composition, this would have implications for their suitability for cell therapy, therefore necessitating further investigation.

Another challenge faced by cell expansion for the purpose of CF cell-based therapy is reports that differentiated cultures established from extended *in vitro* expansion of basal cells often have decreased CFTR ion transport function (Peters-Hall et al., 2018). Lee and colleagues report that although CRC expanded cells have increased CFTR ion transport compared to conventionally expanded cells, short-circuit current (a proxy for CFTR function) still decreased by passage three in CRC expanded cells (Lee et al., 2020). A better understanding of the impact of extended cell expansion on CFTR function is required.

### Transplantation of CFTR-Corrected Regenerative Cells

Basal cells are located within the surface epithelium, adjacent to the basement membrane, and their differentiation leads to the cellular diversity of the airway epithelium. As such, if CFTR-corrected basal cells are to be the basis of a cell therapy, then they would need to be engrafted onto the basement membrane of the airway epithelium. This should replenish the airways with CFTR-corrected ciliated and secretory cells, and ionocytes. Meanwhile, the engrafted basal cells, corrected by integrating *CFTR* addition or mutation-specific gene editing, would retain their capacity for self-renewal to establish a long-term corrected cell population.

Studies of hematopoietic stem cell transplantation show that transplanted cells compete with endogenous bone marrow stroma cells (Abbuehl et al., 2017). Similar concerns have been raised for competition between endogenous and transplanted cells used to repair the lung epithelium. In a competitive repopulation assay, a mixed culture of CF and healthy (non-CF) basal cells were differentiated at air-liquid interface to generate a pseudostratified airway epithelium *in vitro* (Lee et al., 2020). Lee and colleagues found that non-CF cells outcompeted CF donor cells, suggesting that endogenous airway epithelial repair and regeneration is likely to hinder cell engraftment. However, more work is required to fully comprehend and address factors such as these which may impact stable integration of transplanted cells.

The innate barrier properties of the CF airway epithelium–the thick mucus layer and the ciliated pseudostratified multi-layered nature of the epithelial tissue–will likely make the delivery of the corrected basal cells to the basement membrane difficult. An effective cell delivery method will need to first overcome the mucus layer covering the epithelial surface. Whilst aerosolization of stem cell MSCs has demonstrated feasibility as a new method of cell delivery *in vivo* in rabbit airways (Kardia et al., 2014; Kardia et al., 2018; Halim et al., 2019), this technique has not been trialed using basal stem cells. Most studies of basal cell engraftment delivered via intratracheal instillation (Ghosh et al., 2017) or injection (Millerl et al., 2018) use rodent models (Ghosh et al., 2017; Miller et al., 2018). However, these animal models are limited in their capacity to accurately recreate human CF airway pathogenesis. Evidently further testing in appropriate *in vivo* animal models is required to determine an effective approach for cell delivery capable of overcoming the altered mucus barrier in CF. Yet, even if an optimal method is achieved, it is anticipated that disruption of the epithelial cell layer via conditioning or transient injury will be necessary to facilitate effective cell transplantation. Similar strategies have been successful for conditioning of the bone marrow prior to haemopoietic stem cell transplantation (Gyurkocza and Sandmaier, 2014).

Experimental evidence for successful donor cell engraftment in the lungs of animal models has been shown with various injury protocols. Following conditioning with naphthalene, both iPSCs (Miller et al., 2018) and airway basal cells (Ghosh et al., 2017) successfully transplant into mouse airways and persist for up to six weeks. Comparatively, partially stripping the AECs by treatment with polidocanol, an agent that creates a larger site for donor cell engraftment than naphthalene, has enabled basal cells to engraft into live mouse airways and the transplanted cells remained viable for at least three weeks (Farrow et al., 2018a). The long-term effect of these conditioning protocols upon cell engraftment is yet to be examined. In the context of CF, no ideal strategy has yet been developed. To injure the already inflamed and infected lungs of CF recipients might appear counterintuitive**.** The question remains: what is the optimal conditioning scenario that will cause minimal injury, whilst facilitating effective engraftment of corrected cells in sufficient numbers to restore lung function?

## Conclusion

There are clear challenges to the successful translation of gene and cell therapies designed to correct the CFTR defect in the airways. Various novel viral and non-viral therapeutic vectors have advanced to clinical trials; however, no significant clinical benefit has been achieved. The greatest barrier to the success of these gene therapeutics is their delivery to the human airways. Overcoming this barrier will be central to ongoing research and paramount to the achievement of an efficacious *in vivo* CF gene therapy.

Cell-based therapies represent a promising alternative strategy wherein CFTR is corrected *ex vivo*. Yet, questions remain unanswered. Even if regenerative basal cells that are corrected via integrating *CFTR* addition or mutation-specific gene editing can efficaciously transplant at the basement membrane of the airway epithelium, how safe are these *ex vivo-*corrected cells? We know that reprogramming, expansion, and editing increases the probability of tumorigenicity (reviewed in (Berical et al., 2019)). Can basal cells only be expanded to the necessary therapeutic dose at the cost of preserving the transcriptome, epigenome, and differentiation capacity of these basal cells? Long-term investigations are needed to confirm transplanted basal cells are free from mutations, that they are stably engrafted and that CFTR function is retained. Future studies to investigate and improve the findings discussed in this review are required to validate the feasibility of cell-based therapy for treatment of CF.

Moreover, challenges will likely be met in transitioning gene- and cell-based therapies to clinical care. Discussions of the difficulties in developing and sustaining a successful business model for cell-based therapies, and the changes in clinical care necessary to make this potentially transformative therapeutic approach accessible to patients, are ongoing (Elverum and Whitman, 2019). As we continue to forge ahead in this era of personalized medicine, improvements to current CFTR modulator drugs will likely herald increased patient benefit. However, clinical translation of gene or cell-based therapies, though still an ambitious goal, offers future promise of a mutation-agnostic cure for CF.

## Abbreviations

AAV, adeno-associated virus; AAV2, adeno-associated virus serotype 2; AAV6, adeno-associated virus serotype 6; AECs, airway epithelial cells; ASO, antisense oligonucleotide; CF, cystic fibrosis; CFTR, cystic fibrosis transmembrane conductance regulator; CRISPR, clustered regularly interspaced short palindromic repeat; CRC, conditionally reprogrammed cell; iPSCs, induced pluripotent stem cells; IV, immunodeficiency virus; LNPs, lipid nanoparticles; LV, lentivirus vector; mRNA, messenger RNA; MSCs, mesenchymal stem cells; NMD, nonsense mediated decay; TALENs, transcription activator-like effector nucleases; ZFNs, zinc-finger nucleases
